# Physician Perception of Trust and Communication with Asian Patients with Serious Illness and Their Families in the United States: An Exploratory Qualitative Study

**DOI:** 10.1177/26892820251389755

**Published:** 2025-11-04

**Authors:** Cloris Huan, Irene M. Yeh, Francesca L. Troiani, Jennifer Tjia

**Affiliations:** ^1^ Department of Health Policy and Management, University of North Carolina, Gillings School of Global Public Health, Chapel Hill, North Carolina, USA.; ^2^ Department of Supportive Oncology, Division of Adult Palliative Care, Dana-Farber Cancer Institute, Boston, Massachusetts, USA.; ^3^ Department of Population and Quantitative Health Sciences, Division of Epidemiology, UMass Chan Medical School, Worcester, Massachusetts, USA.; ^4^ Harvard Medical School, Boston, Massachusetts, USA.

**Keywords:** Asian, cross-cultural communication, health equity, knowledge, qualitative research, serious illness

## Abstract

**Background:**

Asians in the United States receive less palliative care and enter hospice less than Whites, disproportionately receive more invasive mechanical ventilation, and report less involvement in decision-making with physicians than they would like. Despite the growing literature addressing serious illness in diverse patient populations, communication with Asians is understudied. This study aimed to explore U.S. physician perceptions of clinical interactions with Asian patients with serious illness and barriers and facilitators to physician–patient communication.

**Methods:**

This is an exploratory qualitative descriptive study using semistructured interviews with U.S. physicians who cared for Asian patients with serious illness. We used an inductive content analysis approach to identify themes related to facilitators and barriers to communication between Asian patients, their families, and physicians.

**Results:**

We conducted 10 physician interviews between February and April 2024. Of participants, 50% were White and 50% were Asian, the majority were male, and 50% specialized in palliative care. Three major themes arose: (1) trust cannot be assumed; (2) understanding and honoring the role of family are key; and (3) honoring the patient’s preferences for communication can build trust.

**Conclusion:**

This study is a step in illustrating how a cross-cultural approach to communication needs to align physicians, patients, and families on the process of communication and shared decision-making and not only on the goals for care. Moving toward a cultural adaptive approach can empower clinicians to engage in a trust-building process of inquiry, observation, and understanding of how sociocultural factors impact patient preferences for health care.

## Introduction

Culture shapes how people make meaning of illness and dying.^1–3^ The National Consensus Project^4^ stresses the need to emphasize the cultural aspects of care in palliative care delivery. With increasing global migration, encounters between patients and physicians of different backgrounds are more common, thus increasing the risk of cross-cultural misunderstandings.^1^ Even with the growing literature addressing serious illness in diverse patient populations, there are relatively few studies focused on Asians, especially in the United States, despite being the fastest growing racial or ethnic group in the United States.^5,6^

Marginalized groups of patients, including Asians, report low satisfaction with end-of-life care.^7–9^ Asians report less involvement in decision-making with physicians than they would like,^10^ receive less palliative care and enter hospice less than Whites,^11,12^ and disproportionately receive more invasive mechanical ventilation.^13^ One challenge to addressing disparities for this population is the diversity of traditional norms, values, and religious beliefs among this heterogeneous population who come from East Asia, South Asia, and Southeast Asia. In the United States, Chinese adults comprise the largest subpopulation (22%), followed by Indian (20%) and Filipino adults (17%).^14^

To address care disparities, two bodies of literature have emerged that are relevant to health care delivery for Asian patients. One body of scholarship can be described as “culture specific” and focuses on subgroups of Asians^15,16^ who live in specific locales (e.g., patients in Hong Kong, Mainland China, and Taiwan;^17^ Japanese Americans and Japanese living in the United States;^18^ or South Asians in Canada).^19^ The other body of literature can be described as “culture general” and more broadly addresses how clinicians can improve care for patients from diverse backgrounds.^2,20–23^ While both are critical, several important ideas can become lost when focusing on one or the other. First, palliative and end-of-life care in the United States are historically rooted in White middle-class cultural and religious values,^24^ which creates a dominant culture and assumed approach to communication and care delivery. Second, the idea of culture is often constrained to the notions of race, ethnicity, and country of origin, without adequately acknowledging that other experiences, such as workplace culture or religious practice, can also create cultural subgroups.^23,25^

In 2001, Kagawa-Singer and Blackhall's JAMA article, “Negotiating Cross-Cultural Issues at the End of Life,” addressed the tension of being both “culture-specific” and “culture-general” in end-of-life care.^1^ They accomplished this by inviting readers into the life stories of an African American couple in southern United States and a Chinese American woman from Hawaii, while asking readers to consider the ABCDE framework [attitudes (A), beliefs (B), contexts (C), decision-making styles (D), and environments (E)] for assessing patient and family perspectives on end-of-life conversations.^1^ Since then, much has been written about addressing palliative care disparities for Blacks,^26–28^ but less so for Asians.^6^ Particularly in the United States, the health narrative is shaped by the systematic exclusion and misrepresentation of Asian populations in health research.^7,29–32^ Racialized stereotypes reinforce inaccurate societal impressions that Asians seldom experience disproportionate health disparities,^32,33^ despite Asians’ experiences of racism in health care.^34^

To address the literature gap, we conducted an exploratory qualitative descriptive study to provide a snapshot of physician perspectives on cross-cultural palliative and end-of-life care for Asian patients in the United States. We sought to characterize physicians’ perceptions on barriers/challenges (Theme 1) and facilitators/strategies (Themes 2 and 3) to build trust and effective communication with Asian patients with serious illness. This study contributes to the growing literature about communication with persons with serious illness.

## Methods

Following the Consolidated Criteria for Reporting Qualitative Research guidelines,^35^ we conducted semistructured interviews to characterize physician perspectives on communication with U.S. Asian patients with serious illness and their families. Exploratory-descriptive qualitative research is appropriate to explore a topic with limited coverage in the literature.^36^

### Study sample

Eligible participants resided in the United States, had experience as a practicing clinician with patients who self-identify as Asian and have a serious illness, and were able to provide consent. We used a purposeful sampling approach to recruit a nationwide sample from different states in order to describe the phenomena across diverse locales. We recruited participants by using cold emails and via various public health and physician networks.

### Data collection

#### Interview guide.

The semistructured interview guide was developed based on a literature review^23,37,38^ by the interviewer (C.H.), who worked collaboratively with two palliative care physicians, including a physician-researcher (J.T.) and clinician-educator (I.M.Y.) (Supplementary Table S1). Open-ended and probing questions addressed participants’ personal and professional experiences with Asian patients with serious illnesses, cross-cultural communication, considerations of how “best care” might be defined, and approaches to culturally-adaptive care. The team used interviewer feedback to iteratively refine the guide after the first two interviews. Acknowledging that the term “Asian” encompasses a broad diaspora of people, we did not focus on a specific Asian population in the interview guide.

#### Interview procedure

The interviewer (C.H.) had no prior relationship with participants and conducted one-on-one, semistructured interviews after training and under the guidance of the physician-researcher (J.T.) and clinician-educator (I.M.Y.). C.H. completed interviews on a video-conferencing platform after obtaining verbal informed consent and collecting participant demographics (age, gender, race, and ethnicity) and their specialty. The interviewer kept reflexive notes after each interview. All interviews were recorded, transcribed, and de-identified. Participants provided verbal informed consent prior to participation, which includes the publication of anonymized quotes in this article.

### Data analysis and trustworthiness

We used an inductive content analysis approach^39^ to identify broad content categories and themes related to facilitators and barriers to communication between Asian patients, their families, and physicians. We used MAXQDA (VERBI Software, Berlin, Germany) to support the analysis. Preliminary data analysis occurred simultaneously with data collection, which involved reviewing interview recordings and transcripts to develop an understanding of key concepts. The interviewer (C.H.) used the first two transcripts to create a preliminary codebook. Additionally, the palliative care team members (I.M.Y., J.T.) independently reviewed the first two coded transcripts and, together with the interviewer, consolidated findings into a coding scheme used to categorize excerpts. After completing the majority of interviews, the interviewer (C.H.) and a research assistant (F.L.T.) (hereafter “coders”) used the coding scheme to independently code all interviews. The coders met regularly and consulted with the rest of the team to review, refine, and reconcile coding. Sampling continued until we identified a high degree of redundancy in new interviews, indicative of data saturation.^40^ After all transcripts were coded, the entire research team met to identify key themes. To explore the credibility of the results, we returned the thematic findings to 3 of the 10 participants to check for accuracy and resonance with their experiences.^41^ The Institutional Review Board of the University of North Carolina at Chapel Hill determined this study to be exempt.

## Results

**FIG. 1.**
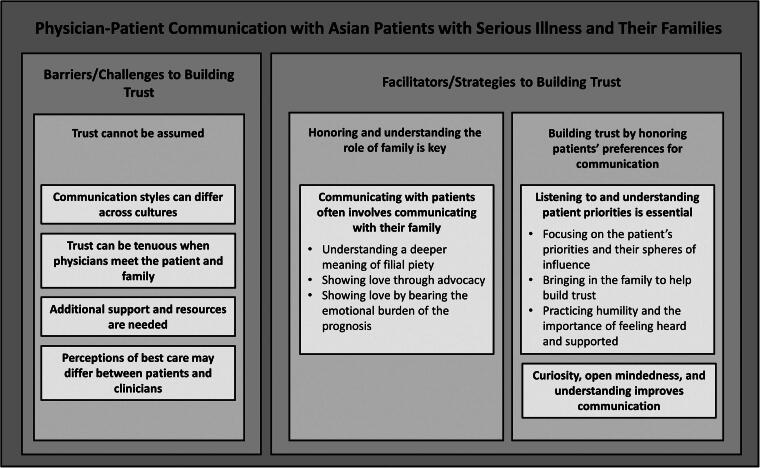
Barriers/challenges and facilitators/strategies for effective communication with Asian patients with serious illness and their families.

We conducted 10 physician interviews between February and April 2024. Interviews lasted 40–75 minutes. Of participants, 50% were White and 50% were Asian, the majority were male, and 50% specialized in palliative care (Table 1). Three major themes arose: (1) trust cannot be assumed; (2) understanding and honoring the role of family are key; and (3) honoring the patient’s preferences for communication can build trust (see Fig. 1). Table 2 provides additional quotes to supplement the results below.

**Table 1.** Participant Demographics

	Total (n = 10)	%
Age		
18–40 years	0	0%
41–65 years	9	90%
>65 years	1	10%
Sex		
Male	6	60%
Female	4	40%
Race		
Asian	5	50%
White	5	50%
Physician specialty		
Hospice and palliative medicine	4	40%
Emergency medicine	1	10%
Geriatric medicine	1	10%
Palliative nephrology	1	10%
Pediatric pulmonology	1	10%
Urologic oncology	1	10%
Psychiatry	1	10%

**Table 2.** Themes, Subthemes, and Representative Quotes in the Context of Communication with Asian Patients with Serious Illness and Their Families in the United States

Theme	Subtheme	Quote
Trust cannot be assumed	Trust can be tenuous when physicians meet the patient and family	*By the time they arrive…they have probably had a lot of medical or healthcare interactions, good and bad. So, my first responsibility, or my first task is building rapport.* (P7) *I just sort of walked into the middle of this long-standing issue.* (P7) *I think this is just a cultural thing. Like I think if we were from her culture or she was from ours, it would make perfect sense. And she just describes pain differently from what we are used to hearing.* (P4) *[T]rust is the most important thing in healthcare interactions. Generally, you have to do more to establish it the more different the person in front of you is culturally from yourself.* (P4) *I think that there is some mistrust of some medical professionals.* (P8) *I’m thinking back to another patient that I had that had so much anger. It was the family. They were just so angry about everything that was happening and they really just wanted somebody to blame and say like, “This is all your fault.”* (P8) *I think trust is really critical. I mean, you have to…from the clinician’s point of view, you have to trust that the patient and caregivers are communicating honestly so that you have a sense…part of your medical decision making has to do with the communication of the symptoms and if you feel like maybe those aren’t being communicated honestly, it’s hard to know how to make good medical decisions. I think going the other way, I think the family has to trust that you understand, as the clinician, you understand their cultural context and their goals and that you are providing them with the best information and care for their family member.* (P6) *I feel like there was always a little bit of a cultural barrier there. I did not understand their background, they did not understand my background. They understood some, and I understood some, but it was always a sense that there was a little bit of a barrier there because of that.* (P3)
	Additional support and resources are needed	*[What types of support or resources do you or have you received about cross-cultural serious illness communication?] None. Not from my department, not from my training, not from my hospital. And we should have more.* (P10) *There are always new things to learn. I think it is helpful people publish research on doing interviews with Asian patients. More updates on how we can be more culturally sensitive.* (P1) *I think the important part is to not lose sight of just, “here is a script or here is a check box. Here are five checkboxes and if you do these things you are culturally sensitive or you are culturally humble and competent and you are good to go.” The reality is it doesn’t quite work like that. The other hard part of this is while a lot of this is outward facing…what we haven’t really talked too much about is what is the inwards piece.* (P9) *I think most of my support came from Asian friends, who knew and could sort of help me understand the culture better.* (P3) *There are a lot of people who actually care and are serious and are interested about communication and cross-cultural communication in the healthcare world…we teach each other and then we learn from each other and we share ideas…It helps a lot.* (P9) *I will bounce things off of my Asian colleagues, and I feel like that is very helpful.* (P1) *If there are ways to share best practices and make sure that this is an ongoing part of continuing education for not just physicians but other healthcare professionals. For example, seminars on cultural competence, cultural proficiency, communication, those sorts of things I think would be very useful.* (P6) *...integration of palliative care and so, like really good serious illness communication in [subspecialty]…is really lacking.* (P8)
	Perceptions of best care may differ between patients and clinicians	*[T]hey don’t know. That’s why they are kind of referring to me, the expert, to decide. But the expert cannot decide without knowing more about the patient.* (P10) *Unfortunately, most of my physician friends don’t make recommendations based on what is good enough to live for that patient. Rather, they just make it based on patient prognosis…depending on patient values and goals, our recommendations for treatment should be different. And yet, that is not routinely practiced.* (P10) *In her mind, she [Chinese patient] specifically did not want a trainee to do this procedure and she wanted somebody with immense experience. It’s kind of how she rated it as best.* (P7) *[W]ell, I am not the doctor. I don’t know how to decide…you’re the one that should be telling me what to do.* (P5) *Well I don’t know. You’re the doctor, you tell me and I’ll do it.* (P7) *What would you do? What would you recommend?* (P5) *[D]o your best. If this was your dad, do what he would want.* (P9) *[S]ometimes people define best care as care by the most knowledgeable specialist or somebody who is [a] higher academic rank or administrative academic position.* (P6)
Honoring and understanding the role of family is key	Communicating with patients often involves communicating with their family	*A lot of times when you say, “well, what is important to you?” this idea where as an individual “you make your own decisions,” Asian patients will often defer to the family.* (P1) *In Western American medicine, you name your decision maker. Who is the person you want to make decisions for you? And they [Asian patients] will look at you like “well the family makes the decision as a group.” You don’t have one person make the decision.* (P1) *[I]t just didn’t feel right to them to deprive their loved one, that they just respected and loved so much, of another chance. They felt sort of like if I do this, maybe she will do better, maybe she will live longer, maybe she will get better.* (P2) *I would say the main thing that characterizes my experience working with Asian patient is this really broad heterogeneity and I guess what I’ve learned is that I can’t make any assumptions walking into a room. I have to try to get some understanding of what the family’s understanding is from the outset and then go from there.* (P6)
Building trust by honoring patient’s preferences for communication	Listening to and understanding patient priorities is essential	*Knowing their prognosis, if that is what they want…[b]ut if they don’t want to know and you tell them, that is not best care.* (P1) *[T]he more I do this, the more I lean on patient preferences and less on my expertise. I offer my expertise…It doesn’t matter how expert I am, if it is not their preference, they are not going to do it anyways.* (P9) *I think when I was younger, I tried to push hard about stopping. As I’ve gotten older, I think sometimes…the most important thing is for the family to feel supported.* (P1) *[U]sing sort of willing family members to kind of work as cultural mediators, I’ve found that helpful.* (P2) *I think we need to respect those kinds of requests of individuals. Again, it’s a negotiation between the two. What we think would be best for them in their minds is not necessarily what they think is best for them.* (P3) *I think we can never spend too much time on talking about the emotions that are driving the concerns…we are just here to provide the type of information that will hopefully be helpful to them, and we can give them recommendations. But just assuring them that whatever decision they make, we will be okay with and supportive of.* (P2) *Sometimes we will say, what is it that I can do for you? Because sometimes we see people who have been to multiple doctors. It’s unclear what the issue really is; I’m not sure what they are seeking. If it is just they are trying to find a tie breaker and they’ve been to two different people and now they want a third person to kind of come down on one side or the other. So, I think…you have to kind of sort out what is most important to them, and then trying to help them achieve the optimal result or outcome for whatever is most important to them is probably their best care.* (P5) *I think more so it’s just trying to be very slow and methodical and simple in my communication and also asking them to sort of tell me back what they heard me tell them just to make sure that I am communicating correctly. It takes more time. But I think if you do take the time, they definitely notice and appreciate it, and that is what builds trust.* (P4)
	Curiosity, open-mindedness, and understanding improve communication	*[N]ot that makes sense to me (the physician), but more importantly, makes sense to the patient and the family.* (P9) *I hear them asking patients, “If your heart stops, do you want us to try to restart it? Or if you stop breathing, do you want us to put you on a breathing machine?” I hear that a lot, and that is okay to ask. What most people fail on is, “So why is that? Why do you say you want to be on a breathing machine? Or you want to have your heart restarted?” I think the most important fact is hidden, that people don’t ask…I think people failed to ask the why question.* (P10) *[L]ooking for the verbal and the nonverbal…emotional cues…tones…what questions are asked…what questions are not asked…how I am being received.* (P9) *I think the most important part is to go into these conversations with an open mind and not so much with an agenda of what the outcome should be. But with an open mind and a real deep interest to try to understand: What it is that worries this patient? What is it that they want to know? How do they want to make decisions as a unit? And when I say family, I don’t necessarily mean blood, but the loved ones.* (P2) *[B]eliefs and values will drive the conversation all day, every day.* (P9) *Why? What makes you say that? And really trying to move deeper to the feelings and fears and legitimate worries that somebody has that is requesting sometimes a care plan that seems non-beneficial to patient or not reasonable in the context of their serious illness. So really trying to understand more and getting them to place where they are comfortable and they don’t feel ridiculed or demeaned or dismissed. So really just showing it, authentic interest.* (P2) *I basically listen and ask questions. And I think that actually builds a kind of trust. They believe, when I’ve done that, that I am truly interested in them and their background. I don’t feel a necessity to talk about my own background, but at the same time I am not trying to suggest to them that I know a lot about you. I just admit, there are things that I just don’t know and you are going to have to help me with this.* (P3)

### THEME 1: Trust cannot be assumed

#### Communication styles can differ across cultures

Participants noted that although trust is universally beneficial to care, trust-building strategies vary by culture and personal experience.

*…if I had to sort of try to identify some of the differences, though not unique to Asian patients, is probably more use of metaphors and…indirect forms of communication, so oftentimes there is more relying on context. There is…I was going to say, need to build trust, but that is not right. The reason I pause is because I think that [trust] is important for all patients. But…the way that you build trust oftentimes for Asian patients may be different than other patients*. (P9)

Silence and mismatch of communication styles complicated encounters.

*[Y]ou can be in a meeting with an Asian family, and everyone is sitting there very quiet, and you think everyone is agreeing with you. Then, it turns out they are super mad, but no one is saying anything…it’s sort of like a respect thing. So, you have no idea.* (P1)

#### Trust can be tenuous when physicians meet the patient and family

Participants recognized that patients’ previous experiences with health care impacts their receptiveness to health care interventions and communication.

*If this is the fourth time that they are having this conversation, they have already been kind of traumatized. And they feel that we are not listening to them, and they have to advocate more and more.* (P2)

Approaches to conversations are culturally bound. Some physicians described the U.S. health care systems’ approach to serious illness conversations as failing to capture the needs of marginalized populations.

*[T]he way conversations about serious illness was…formed and built was by White people…it was informed by what works when you talk to White patients about their illness, their understanding of their illness...a lot of the questions we were taught to ask...are informed by the fact that it was predominantly White people who…taught us this.* (P2)

#### Additional support and resources are needed

Several participants revealed low confidence in their ability to navigate cross-cultural serious illness conversations.

*I have a hard time feeling confident that I understand someone else’s beliefs and values, though [we] always discuss what those are.* (P6)

At times, unease was amplified in situations where the patient and family members had prior negative health care experiences.

*I feel like sometimes I am just another link in the chain of: this has been difficult, we haven’t felt listened to, this is another person who doesn’t want what I want.* (P2)

Participants felt that their unease was exacerbated by a lack of training on how to navigate cross-cultural serious illness conversations. Few participants received formal training for cross-cultural communication in the United States.

*I have not received any training or skills workshops or really any tips, like what I should be looking out for…* (P8)

Nor did they receive any information about communicating with Asian populations.

*Not a lot about Asian populations…I think [we] probably could use more.* (P5)

Most participants learned about cross-cultural communication techniques and approaches for Asian patients with serious illness through self-initiated opportunities (e.g., conversations with colleagues and friends) and resources (e.g., journal articles).

*Just in my personal life, and in my professional life with my colleagues, the literature, in meetings, and also just talking through cases.* (P1)

Overall, participants wanted more resources about cross-cultural communication with Asian patients with serious illness and their families.

*In general, if there was more teaching about communication and…culturally sensitive communication beyond palliative care…I think that would be very helpful…there is still a big gap.* (P2)

#### Perceptions of best care may differ between patients and clinicians

##### Physician expertise confers trust.

Some physicians noted that a lack of interpersonal trust can lead to patients’ reliance on hierarchy.

*[A] lot of our patients don’t really have a personal trust [of clinicians]. They’re kind of trusting more in the fact that we’re on faculty at [redacted].* (P5)

Similarly, social hierarchies (e.g., medicine judged as a respectable profession) can influence communication with physicians viewed as “experts.” Participants noted that Asian patients may seek out and defer to professional expertise on what care plan options to pursue.

*[D]epending on the culture, I think patients and families perceive physicians to be definitely more in higher power than they are.* (P9)

*Whatever you think doc…you know better than I do.* (P8)

However, differences in the expectation and perception of “best care” between physicians, patients, and families can make it challenging to provide high-quality care that aligns with patient preferences and fulfills patient and family’s expectation of power distance.

*[T]he challenge is how can you get their perspective and what is important to them while also sort of fulfilling the role that they expect. Which is that you guide them and tell them what to do.* (P4)

Participants also warned that if patients are only following recommendations due to the cultural belief of physicians as authority figures, and if physicians reinforce this power dynamic, it may hinder care.

*[I]f culturally they feel like they have to do what the doctor says but really, they are not into it…they are doing it because you are an authority figure. I think you are also doing the patient and the family a disservice.* (P9)

### THEME 2: Honoring and understanding the role of family are key

#### Communicating with patients often involves communicating with their family

It was difficult when patients involved or deferred clinical communication to family members.

*[Y]ou have to do both dances, two different levels of communication…in the same encounter...I am having different conversations with the family and the patient.* (P8)

*I guess the way we are trained…[is] to be very patient-centric…But when the patient themselves opts out, that makes that really difficult.* (P7)

##### Understanding a deeper meaning of filial piety

Filial piety is perceived as an Asian phenomenon, traditionally linked to duty and obligation. However, filial piety can also represent a practice of love through acts of service.

*[F]ilial piety is a big value in Asian culture.* (P4)

*[F]or Asian families, it’s a way of wanting what is best for them. It’s a way of showing I care.* (P9)

##### Showing love through advocacy

Some physicians inferred that Asian patients and families adapt their health care-related behavior and decision-making to demonstrate love and duty, and thus seek out the “best care” by seeking additional information, care, and/or services.

*[T]he way of showing love and support is through seeking more care, more aggressive care….I think the disconnect is around giving up hope…the family feeling like if we pursue more palliative care, then we are giving up on our loved one or we are sort of not supporting them enough.* (P2)

##### Showing love by bearing the emotional burden of the prognosis

Participants noted that Asian families may express a protective, advocating approach to patient care to preserve their loved one’s fortitude. Physicians observed this belief through nondisclosure, where patients were unaware of the full extent of their condition.

*[A] family will say, “I don’t want my mom to know she has cancer or…is going to die. Because that will just kill her spirit.”* (P1)

### THEME 3: Building trust by honoring patients’ preferences for communication

Participants recognized that differences between physician, patients, and family cultures, beliefs, and values can create communication challenges.

*[W]hen you are dealing with families and patients that are outside of your culture…it is a challenge.* (P7)

Facilitators of trust were noted to include: 1) listening to and understanding patients’ priorities and 2) demonstrating curiosity, open-mindedness, and understanding.

#### Listening to and understanding patients’ priorities are essential

##### Focusing on the patient’s priorities and their spheres of influence

Participants shifted priorities toward supporting a patient’s well-being by meeting the patient where they are and forming care plans around the patient’s priorities.

*It’s whatever you can do to achieve that alignment of their hopes and their wishes as it matches their values and in the context of reality. I think it incorporates the spheres that are most important to them. That sphere might be family, it might be their own personal sphere, it might be spirituality….*(P8)

I don’t try to force them to decide. (P10)

##### Bringing in the family to help build trust

Participants noted that, among Asian patients, the inclusion of family members in serious illness discussions is vital.

*I think if you don’t have the family involved, it often can fail…our best allies in working with patients is to work with their families.* (P3)

##### Practicing humility and the importance of feeling heard and supported

Participants noted that identifying and integrating patient and family core values and beliefs into the care plan build trust.

*I used to be quite…certain that I had all the answers and that…my recommendations were the most valid. But as I’ve gone on in my practice, I have decided that sometimes the patient just needs to be heard and realize that I am going to try to help them even if I don’t think that this is the right medicine for them...*(P7)

#### Curiosity, open-mindedness, and understanding improves communication

To build trust and ensure that Asian patients and families feel heard and seen, physicians clarified that decision-making should ultimately center on patient and family preferences.

*[E]ntering these conversations with true curiosity, true need to understand, and really trying to listen where they are coming from, and not so much imposing where I need to get in these conversations.* (P2)

*[T]he data is there. You just have to be willing to see it.* (P9)

## Discussion

Communication between clinicians and patients with serious illness often involves high-stakes encounters. This qualitative study explores the perspectives of a nationally drawn sample of clinicians about conversations with Asian patients with serious illness in the United States. Unseen in prior studies, participants noted that trust in clinician–patient encounters could not be assumed but earned through the development of a relationship, and that patient or family silence in conversations does not surmise agreement. Our findings shed light on the complexities of the family’s role in communication and advocacy and underscore the challenges that clinicians report in struggling to provide goal-concordant care across cultures.

What is seldom acknowledged in cross-cultural communication studies is that one culture is typically dominant and the other nondominant. Explicitly stating that a hierarchy of cultures exists can help us acknowledge that the person from the nondominant culture (e.g., patient/family) is often in the position of having to respond by choosing to “assimilate, accommodate, or separate” from the communicator from the dominant culture, according to the communication theorist Mark Orbe.^25^ His Co-Cultural Communication Theory^25^ can help us think about mismatches in expectations, where physicians may expect patients to have an “assertive” communication style when patients may have a “nonassertive” or even an “aggressive” communication style.

Misinterpretations of patient and family motives based on their actions are not surprising when one considers that the quote “Your silence gives consent” is attributed to Plato, a foundational giant of Western culture. The prior literature acknowledges that Asians often use indirect communication strategies,^42–44^ which rely on inferred meaning. Nodding and smiling to show respect for authority^45,46^ are gestures that may be misunderstood as signs of agreement if different meanings are inferred. Moreover, if the patient or family member does not agree with the treatment plan, disagreement may appear indirectly as resistance as it may feel inappropriate to openly disagree with “authority.”^47^ Continued dissonance in patient–clinician communication styles and understanding of the patient’s health care wants and needs erodes trust.^1,17,48^

This finding highlights the importance of the process of involving families in a meaningful way when such involvement is preferred by the patient and feasible. Throughout our study, conversations on “filial piety” arose. However, we are concerned that the limited definition of filial piety as duty and obligation perpetuates the misrepresentation of Asian populations in health research and facilitates the oversimplification of individual health preferences.^7,29–32^ Our analyses go beyond the filial piety label to illustrate how families show love through advocacy, a finding consistent with a study among Korean patients where a key theme was that families bear the emotional burden of illness by “becoming a ballast in the journey toward death.”^17^ Dishonoring this meaningful family act can be a threat to physician–patient trust.

Furthermore, the disclosure of a terminal prognosis is regarded as imperative in end-of-life care in Western medicine, but truth-telling is not a globally shared moral position.^2,49^ The U.S. health care system’s reliance on the shared decision-making model is grounded primarily on the ethical idea of individual autonomy.^49^ In contrast, beneficence and nonmalfeasance are core concepts within the ethical model that is more dominant in Asian cultures, consistent with the collectivist values of relational autonomy.^49,50^ Family involvement would then be considered much more than “filial piety” if defined solely as duty and obligation. It should be also acknowledged that including family is broadly important to providing palliative care to patients across various cultural backgrounds, but effective approaches may vary depending on the cultural context.^20,51–56^

Taken together, we ask the reader to consider that the examples of cultural differences highlighted in our study (e.g., communication style, inclusion of family priorities) illustrate how communication, especially in the setting of serious illness, can collide across cultures. Consideration of culture would go beyond knowing about belief systems, values, and expectations^22^ but how it impacts the six core functions of health communication, including fostering the patient–family–clinician relationship, exchanging information, responding to emotions, managing uncertainty, making decisions, and enabling patient/family management.^25,57^ A key theme of our study is the importance of the need for physicians to *listen to and learn* from patients and families about their process and communication preferences.^23^ We argue that this is more than just “cultural humility,” defined as the process of self-reflection to understand and respect cultural differences. Instead, our findings support a need for the clinician communicator to actively flex one’s own cultural framework to become more effective communicators. Our findings suggest that the process starts with “true curiosity” and “really trying to listen to where they are coming from” in order to build trust and then requires clinicians to have practical communication skills that enable them to understand a patient’s desired process of how to communicate and make decisions. This culturally adaptive approach^37^ bridges the tension of being both “culture-specific” and “culture-general” in serious illness communication. Cross-cultural education has historically focused on the cognitive activities of asking questions and learning cultural traits that could easily become stereotypes, with less emphasis on attitude and skills. Cultural adaptivity is a process of inquiry, observation, and understanding how sociocultural factors impact health-related behaviors and preferences, all in the service of becoming a more effective patient and family-centered communicator.^58^ An adaptive approach is consistent with Bettencourt’s argument that we need “less knowledge” and “more skills.”^58^ The participants in this study stressed how resources and additional training to equip clinicians with this method in the United States are sorely needed.

Limitations of this study include a smaller sample size that does not encompass the range of locations, socioeconomic statuses, and cultural differences across the country. This study also utilizes physician perspectives to characterize patient–provider communication in the United States. Further study is needed to understand patient and family experiences. Nevertheless, the study population included individuals from varying locations, health care facilities, cultures and ethnicities, clinical experience, and specialties. Finally, while we did not specify a particular Asian subgroup in the interview guide, we acknowledge that participants’ examples trended toward patients from East Asian populations.

In summary, culture affects both the content and process of communication and decision-making. Our findings from physician interviews about Asian patients in the United States highlight the need for clinicians to be able to communicate in a way that gains alignment on the content for care goals, process on how to discuss care goals, and includes both patient and family priorities on the journey. Our study provides valuable insights into physicians’ adaptive strategies from respondents’ narratives: listen carefully, remain open-minded, be supportive and offer options, leverage engaged family members, use simple and clear communication methods, and take time. Further work to understand and develop clinicians’ skills in these areas is needed to get us closer to reducing disparities in palliative and end-of-life care.
