# Supplementary material for: Physician Perception of Trust and Communication with Asian Patients with Serious Illness and Their Families in the United States: An Exploratory Qualitative Study
Source: Palliat Med Rep. 2025 Nov 4;6(1):542–53. doi: 10.1177/26892820251389755 (PMC12670691; doi:10.1177/26892820251389755)
Supplement: Supplementary Table S1 [file 26892820251389755_supplementary_table_s1.docx]

**SUPPLEMENTAL MATERIAL**

**eTable 1. Reflexive Stance of Study Authors**

| **Author** | **Reflexive Stance** |
| --- | --- |
| CH | A female, second-generation Chinese-American without clinical training or personal experience with serious illness. At the time of the study, CH was a senior undergraduate student. CH holds a bachelor’s of science in public health degree in health policy and management. |
| JT | A female, second-generation, physician-researcher of mixed Southeast Asian-Chinese background trained in geriatric palliative care and epidemiology. JT has personal and clinical experience in the care of seriously ill Asian patients and their caregivers. JT received training in qualitative methods and has published numerous qualitative manuscripts. |
| IMY | A female, second-generation Taiwanese American, internal medicine and palliative care trained senior physician with extensive clinical, administrative, and medical educational experience advocating for health equity in palliative care. IMY has clinical experiences in the care of seriously ill Asian patients and their caregivers. |
| FLT | A female, who self-identifies as White, and has prior experience working with nursing home residents. FT has a bachelor’s degree in biology with a certificate in gerontology. |
